# Polydatin effectively attenuates disease activity in lupus-prone mouse models by blocking ROS-mediated NET formation

**DOI:** 10.1186/s13075-018-1749-y

**Published:** 2018-11-12

**Authors:** Pan Liao, Yi He, Fangyuan Yang, Guihu Luo, Jian Zhuang, Zeqing Zhai, Lili Zhuang, Zhuomiao Lin, Jiehuang Zheng, Erwei Sun

**Affiliations:** 1 Department of Rheumatology and Immunology, The Third Affiliated Hospital, Southern Medical University, No. 183, Zhongshan Avenue West, Tianhe District, Guangzhou, 510630 China; 2 Institute of Clinical Immunology, Academy of Orthopedics Guangdong Province, Guangzhou, China; 3 School of Pharmaceutical Science, Southern Medical University, Guangzhou, China

**Keywords:** Neutrophil extracellular trap, NETosis, Reactive oxygen species, Systemic lupus erythematosus, Polydatin

## Abstract

**Background:**

Neutrophil extracellular trap (NET) formation has been described to be closely involved in the pathogenesis of systemic lupus erythematosus (SLE). In this study, we aimed to investigate the effect of polydatin (PD) on NET formation and its effects on disease activity in lupus-prone mouse models.

**Methods:**

In vitro, neutrophils from SLE patients and healthy people stimulated with phorbol 12-myristate 13-acetate (PMA) or phosphate-buffered saline (PBS) were treated with PD, and reactive oxygen species (ROS) production and NET formation examined. In vivo, pristane-induced lupus (PIL) mice were treated with vehicle, PD, mycophenolate mofetil (MMF) or cyclophosphamide (CYC) while MRL/lpr mice were treated with vehicle or PD. Proteinuria, serum autoantibodies, ROS production, NET formation and kidney histopathology were tested.

**Results:**

Consistent with previous findings, blood neutrophils from SLE patients showed increased spontaneous NET formation. Both in vivo and in vitro, PD treatment significantly inhibited ROS production and NET release by neutrophils. In MRL/*lpr* mouse model, PD administration reduced the proteinuria, circulating autoantibody levels, and deposition of NETs and immune complex in the kidneys. In addition, PD treatment ameliorated lupus-like features in PIL mice as MMF or CYC did.

**Conclusions:**

PD treatment inhibited ROS-mediated NET formation and ameliorated lupus manifestations in both PIL mice and MRL/*lpr* mice. These results highlight the involvement of NETosis in SLE pathogenesis and reveal that PD might be a potential therapeutic agent for SLE or other autoimmune diseases.

## Introduction

Systemic lupus erythematosus (SLE) is a chronic progressive autoimmune disorder manifested by autoantibody overproduction and multi-organ involvement [1]. It is prominently prevalent in African, Asian, Hispanic and American patients [2] and mostly occurs in young women [3]. Over the past decades, most studies on SLE have focused on the dysregulation of adaptive immunity [4, 5]. Although abnormalities of B and T lymphocytes are considered to play central roles in the pathogenesis and development of SLE [6], the active role of the innate immune system in induction of autoimmune response in SLE is equally important. Neutrophils, the most abundant sensory and effector cells in the innate immune system, have attracted more attention in recent years [7].

NETosis is a specific form of cell death of neutrophils, during which the chromatin decondenses and is released into extracellular space with cytoplasmic proteins, forming neutrophil extracellular traps (NETs) [8]. Indeed, increased NET deposition has been found in the skin and kidneys of SLE patients [9]. Neutrophils from SLE patients have been shown to exhibit an increased propensity for NET formation [7, 10]. Owing to the presence of DNase1 inhibitors or anti-NET antibodies, most SLE patients show defective NET degradation. The impaired clearance of NETs has been reported to be closely associated with renal involvement [11]. In addition, NETs activate plasmacytoid dendritic cells and NLRP3 inflammasome in macrophage to release inflammatory cytokines including type I interferons, interleukin (IL)-1 and IL-18, further amplifying inflammatory and immune responses [12–14]. More importantly, many studies have indicated that NET formation causes tissue damage and vascular injury in patients with SLE [9, 15, 16]. Taken together, NETosis plays an important role in the pathogenesis and development of SLE. To date, efforts have been paid to examining the underlying molecular mechanisms of NETosis and found that ROS is necessary for NETosis [17]. A ROS scavenger, N-acetyl cysteine (NAC), has been reported to significantly prevent NET formation [18, 19]. In particular, spontaneous NETosis could also be inhibited by Mito TEMPO, a specific scavenger for mitochondrial ROS production [10].

Polydatin (PD) is an active stilbene compound extracted from a traditional Chinese herb (*Polygonum cuspidatum*). It has gained particular interest because of its strong anti-oxidative effects by blocking the generation of ROS [20, 21]. PD has just been accomplished a phase II clinical trial for irritable bowel syndrome (IBS) and identified to ameliorate IBS-related manifestations [22]. However, to our knowledge, no researches have examined whether PD inhibits the NET formation or has a therapeutic effect on SLE. Therefore, the purpose of this study was to investigate the potential effects of PD on NET formation and determine whether PD is effective in lupus-prone mouse models.

## Materials and Methods

### Mice

Female BALB/c mice (6–8-weeks) were obtained from Experimental Animal Center of Southern Medical University (Guangzhou, China). Female MRL/*lpr* mice (6–8-weeks old) were purchased from SLAC Laboratory Animal Company (Shanghai, China). All animals were maintained under specific pathogen-free conditions in the laboratory animal center of Southern Medical University (Guangzhou, China).

The protocols of animal experimentation were approved by the Ethics Committee of The Third Affiliated Hospital, Southern Medical University (No. L2017032).

### Pristane-induced lupus (PIL) and MRL/*lpr* mouse models

PIL was induced by a single intraperitoneal injection of 500 μl pristane (Sigma-Aldrich, St. Louis, MO, USA) in female BALB/c mice at 6–to 8 weeks of age and followed over 26 weeks. Mouse 24-h urine was collected every 2 weeks, beginning when the mice were at the age of 12 weeks. Treatments started when proteinuria was evident in all Balb/c mice, and continued for another 16 weeks. In clinical practice, mycophenolate mofetil (MMF) and cyclophosphamide (CYC) are two common immunosuppressive drugs for lupus nephritis. Therefore, these two drugs were selected as positive controls. PIL mice were divided into four groups with nine mice in each group, named PIL model, PD, CYC and MMF groups respectively. In the PIL model group, mice received the same amount of vehicle solution (dehydrated alcohol-propylene glycol-Na2CO3-NaHCO3 buffer (pH 8.5)) for PD instead. Six normal mice were also treated with vehicle only as control. In PD group, mice were injected intraperitoneally with a dose of 45 mg/kg PD (a generous gift from Professor Ke-seng Zhao) every day, as reported previously [23, 24]. In CYC group, mice were administrated with CYC (1.8 mg/mouse, weekly, obtained from Baxter Oncology GmbH, Halle, Germany) by intraperitoneal injection. In MMF group, mice were treated with MMF (100 mg/kg, purchased from Huadong Pharmaceutical Co., Ltd, Hangzhou, China) by daily oral gavage [25, 26]. MRL/*lpr* mice were divided into two groups and then received PD (45 mg/kg) or an equal volume of vehicle by daily intraperitoneal injection for 8 weeks.

### Isolation of neutrophils from human and mice

The human blood samples (5 ml each) were obtained from healthy donors and SLE subjects from The Third Affiliated Hospital, Southern Medical University. Human blood neutrophils were isolated by dextran sedimentation and centrifugation [27]. For the isolation of mouse neutrophils, femurs and tibias of mice were removed and bone marrow collected. The bone marrow-derived neutrophils were obtained using mouse bone marrow neutrophil cell isolation kit (TBD Sciences, Tianjin, China), following the manufacturer’s instructions.

### Cell viability assay

Human blood neutrophils were incubated in the absence or presence of various concentrations of PD (50, 75, 100, 125 and 150 μg/mL) and the cell viability tested by Cell Counting Kit (CCK-8) (Dojindo, Beijing, China) following the manufacturer’s instructions.

### ROS assessment

Neutrophils isolated from human blood or mouse bone marrow were incubated with 10 μM 2′,7′-dichlorodihydrofluorescein diacetate (DCFH-DA) (Sigma-Aldrich) in Roswell Park Memorial Institute-1640 (RPMI-1640) at 37 °C for 20 min. After three washes with RPMI-1640, neutrophils were transferred into a 96-well plate (1 × 10^6^ cells/well in 200 μl). Then they were stimulated with 25 nM PMA (Sigma-Aldrich) for 30 mins (some cells were pretreated with PD for 1 h), and the fluorescence intensity measured by SpectraMax M3 (Molecular Devices, San Jose, CA, USA) fluorescent plate reader at 485 nm (excitation)/520 nm (emission).

### Quantification of NETs

To quantify spontaneous and phorbol 12-myristate 13-acetate (PMA)-induced NETs, neutrophils isolated from human blood (3 × 10^5^cells/well in 200 μl) were seeded into black, flat-bottomed, 96-well plates and cultured with phosphate-buffered saline (PBS) or 25 nM PMA in a humidified incubator at 37 °C with CO _2_ (5%) for 4 h. Extracellular DNA was stained with the membrane-impermeable DNA-binding dye-SYTOX green (Thermo Fisher Scientific, Waltham, MA, USA). The plates were analyzed using SpectraMax M3 fluorescent plate reader (Molecular Devices) at excitation 485 nm and emission 520 nm. The spontaneous NET release by mouse bone marrow-derived neutrophils was measured similarly as NET formation tested in human blood neutrophils.

### Immunofluorescence analysis of NET formation

Human blood neutrophils were pretreated with or without PD for 1 h. These prepared neutrophils were placed on cytospins and stimulated with 25 nM PMA or PBS for 4 h. Alternatively, bone marrow-derived neutrophils from PD-, solvent-, or saline-treated mice were directly placed on cytospins and cultured in the absence of serum for 4 h. And then all those neutrophils were fixed with 1 ml PBS/1% paraformaldehyde. After blocking with 5% fetal bovine serum, the cytospins were stained with rabbit anti-myeloperoxidase (MPO) antibody (Abcam, catalog ab208670, Cambridge, UK) in combination with a second Cy3-conjugated goat anti-rabbit immunoglobulin G (IgG) antibody (Servicebio, catalog GB21303, Boston, MA, USA). The DNA was visualized by 4′, 6′-diamidino-2-phenylindole (DAPI) (Thermo Fisher Scientific). After being mounted, specimens were analyzed using a fluorescence microscope.

### Proteinuria detection

All mice were placed in metabolic cages for 24 h urine collection. The concentrations of proteinuria were detected by Bradford Protein Quantification Kit (Bioteke Corporation, Beijing, China). The following scale was used for assessment: 0 score = 0–30 mg/dl; 2 score = 100–300 mg/dl; 3 score = 300–2000 mg/dl; 4 score>2000 mg/dl.

### Quantification of autoantibodies in serum

The serum levels of anti-dsDNA antibodies and anti-Sm antibodies were measured with enzyme-linked immunosorbent assay (ELISA) kits in accordance with the manufacturer’s instructions (Cusabio Life Science Inc., Wuhan, China).

### Flow cytometry analysis

To test the percentage of necrotic cells in peripheral blood-derived neutrophils from mice, heparinized blood samples were lysed with Lysing Buffer (BD Biosciences, San Jose, CA, USA) to eliminate erythrocytes. And then single-cell suspensions were stained with FITC-labeled Ly6G (BD Biosciences) and propidium iodide (BD Biosciences). Flow cytometry acquisition was performed with the FACSVerse™ (BD Biosciences) and the stained cells analyzed with flow cytometry software.

### Assessment of histopathological changes and IgG deposition in kidneys

Kidney tissues were fixed with 10% formalin and embedded in paraffin for preparation. And then these paraffin-embedded sections were stained with hematoxylin and eosin (H&E). Histopathological changes in kidneys were reviewed by the pathologist who was blinded to the experimental information. Lupus disease activity was graded by the Austin score, as described previously [28].

For assessment of mouse IgG deposition in kidneys, frozen sections were analyzed. The frozen sections were blocked with 5% fetal bovine serum and then stained with Alexa Fluor 555-conjugated goat anti-mouse IgG (Abcam, catalog ab150114). Twenty-five glomeruli per kidney of mouse were examined by two observers in a blinded manner. Based on the mean intensity of fluorescence, the scores for IgG deposition were estimated on a scale of 0–3, according to previously described methods [29].

### Assessment of NET formation in kidneys

Formalin-fixed and paraffin-embedded kidney sections were blocked with 5% fetal bovine serum, and then the MPO-stained with rabbit anti-myeloperoxidase antibody (Abcam, catalog ab208670) in combination with a second Cy3-conjugated goat anti-rabbit IgG (H + L) antibody (Servicebio, catalog GB21303). The chromatin was stained with DAPI (Thermo Fisher Scientific). Colocalization of myeloperoxidase and chromatin was recognized as NETs. Ten glomeruli per animal were analyzed for NET staining and the percentage of NET-stained glomeruli calculated.

### Statistical analysis

Data are represented as the mean ± SD. According to data distribution, statistical analysis was performed by Student’s *t* test, one-way analysis of variance or the nonparametric Wilcoxon rank-sum test. *P* values < 0.05 were considered significant differences. All data were analyzed by SPSS software (version 20.0) (IBM Corp., Armonk, NY, USA).

## Results

### SLE patients showed enhanced spontaneous NET formation

**Fig. 1:**
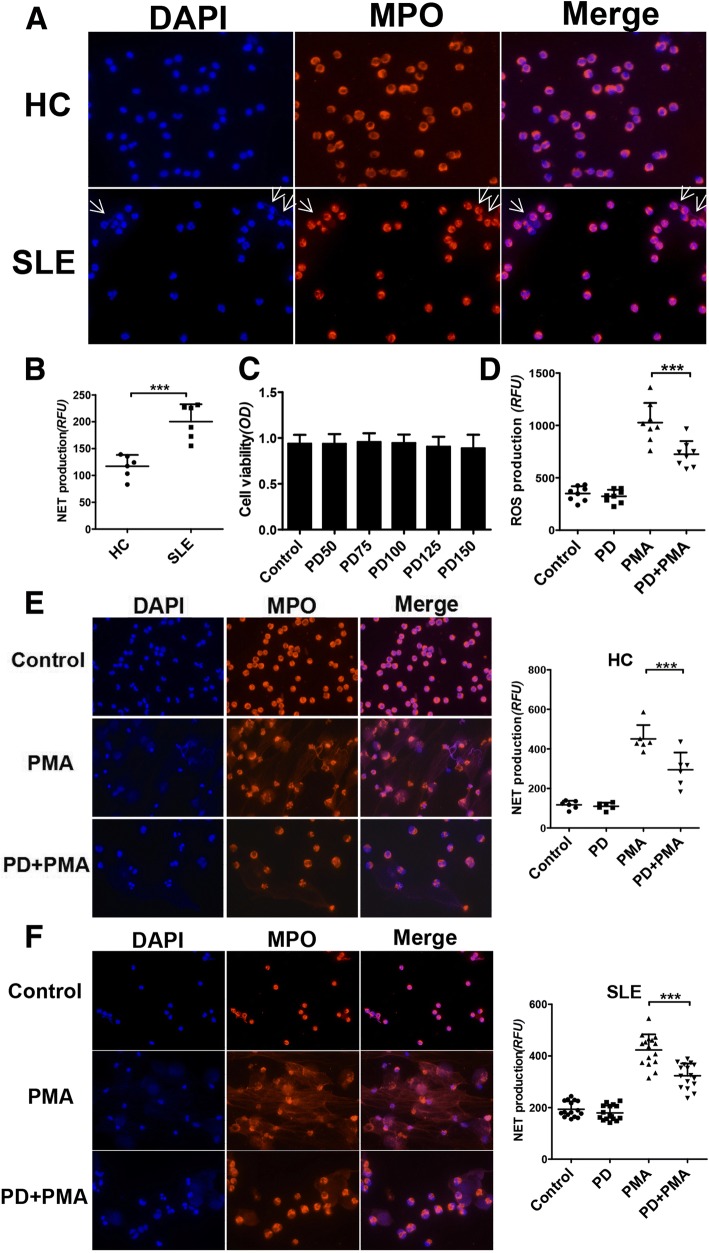
SLE patients displayed increased spontaneous NET formation and PD inhibited PMA-induced NET formation by blocking ROS production. **a** Neutrophils were isolated from peripheral blood of SLE patients and healthy controls (HC) and then incubated with RPMI-1640 for 4 h. Representative fluorescence microscopic images showing NETs that contained DNA (*blue*) and MPO (*red*) (× 400). *White arrows* indicated NETs. **b** Neutrophils from SLE patients spontaneously released increased NETs. NET formation was quantified by SpectraMax M3 fluorescent plate reader. **c** Cell viability of PD-treated neutrophils. Cell viability of neutrophils was measured by CCK-8 assay after stimulation of PD (50, 75, 100, 125 and 150 μg/mL). **d** Neutrophils were isolated from SLE subjects and pretreated with PD for 1 h, followed by stimulation with PMA for 30 min. Cells were stained with DCFH-DA, and the fluorescence intensity was measured. **e**–**f** Neutrophils prepared from healthy controls (HC) and SLE patients, were treated with PD for 1 h and then exposed to PMA for 4 h. NET formation was quantified as described in Materials and Methods (*right*). Representative fluorescence microscopic images of NET formation from HC and SLE patients are shown (× 400) on the left, respectively. For all experiments, data were shown as mean ± SD, *p < 0.05; **p < 0.01; ***p < 0.001. *DAPI* 4′, 6′-diamidino-2-phenylindole, *HC* healthy control, *MPO* myeloperoxidase, *NET* neutrophil extracellular trap, *PD* polydatin, *PMA* phorbol 12-myristate 13-acetate, *SLE* systemic lupus erythematosus

Previous studies have indicated that neutrophils from SLE patients exhibit high potential for spontaneous NET formation. Here, to test spontaneous NET formation, neutrophils were isolated from SLE patients and healthy controls (HCs), and then cultured in RPMI-1640 for 4 h. Indeed, we found that neutrophils from SLE patients spontaneously released higher levels of NETs than healthy control neutrophils (Fig. 1a and b).

### PD significantly inhibited the PMA-induced NET formation by blocking ROS production

We first checked the cytotoxicity of PD on the neutrophils by CCK-8 assay and found that PD had no cytotoxic effect on neutrophils even at a concentration as high as 150 μg/ml (Fig. 1c). Based on this result in combination with other previous studies [25], we selected PD 100 μg/ml for the following vitro experiments.

Many studies have demonstrated that ROS overproduction plays a crucial role in NET formation. PMA is a strong inducer of NET formation dependent on ROS production. To identify whether PD could reduce intracellular ROS production, neutrophils were treated with PD for 1 h and then stimulated with PMA for 30 min. Treatment with PD significantly suppressed PMA-stimulated ROS generation (Fig. 1d).

Next, to investigate the effects of PD on NET formation, neutrophils were pretreated with PD for 1 h, followed by stimulation with PMA for 4 h. We found that PMA stimulation significantly increased NET formation by both SLE neutrophils and HC neutrophils, while PD treatment could markedly suppress the PMA-induced NET formation in both groups (Fig. 1e and f). Overall, these results indicated that PD treatment inhibited PMA-induced NET generation perhaps by blocking ROS production.

### PD treatment alleviated lupus-like features in MRL/*lpr* mice

**Fig. 2:**
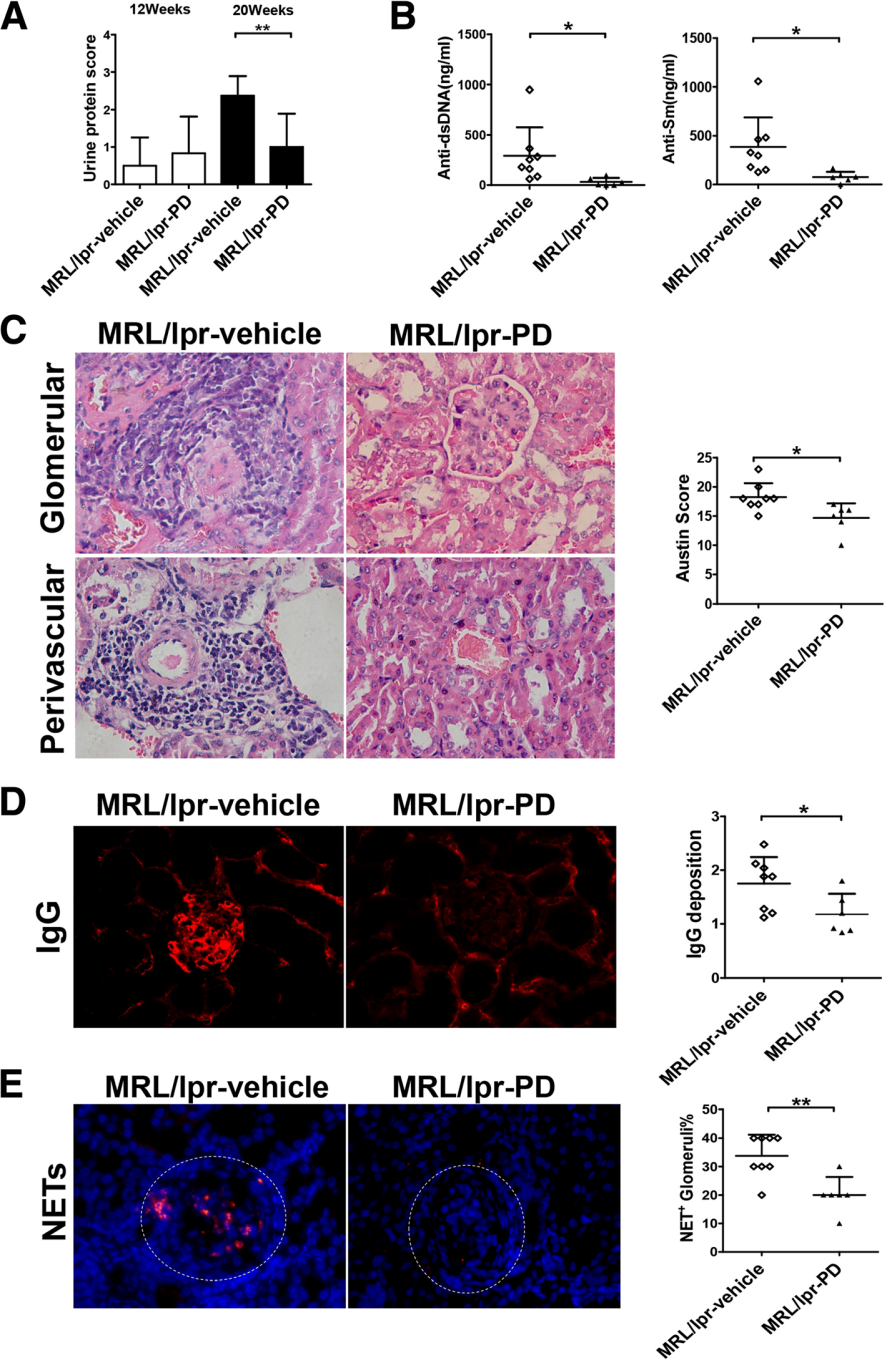
PD markedly reduced autoantibody production, renal disease activity and NET deposition in MRL/*lpr* mouse model. MRL/*lpr* mice were treated with vehicle or with PD (45 mg/kg) by daily i.p. injection for 8 weeks. **a** The proteinuria concentrations were measured by Bradford Protein Quantification Kit and then the urine protein score assessed as described in Materials and Methods. **b** The levels of anti-dsDNA antibodies and anti-Sm antibodies were examined at the age of 20 weeks. **c** On the *left,* representative H&E staining of glomerular and renal vascular lesions in kidneys was shown (× 400). On the *right*, Austin scores of kidneys were determined. **d** On the *left,* representative staining of IgG deposition (*red*) in glomeruli of kidneys was shown (× 400). On the *right*, the average score of IgG deposition was calculated as described in Materials and Methods. **e** NET formation in kidneys was determined as colocalization of DNA (*blue*) and MPO (red). Representative fluorescent images were shown (× 400) on the left. Ten glomeruli per animal were examined for NET staining and the percentage of NET-stained glomeruli per animal was calculated and shown on the right. NET^+^ Glomeruli% = positive NET-stained glomeruli/total glomeruli per field. For all experiments, vehicle-treated MRL/*lpr* mice, *N* = 8; PD-treated MRL/*lpr* mice, *N* = 6; data were shown as mean ± SD, *p < 0.05; **p < 0.01; ***p < 0.001. *IgG* immunoglobulin G, *NET* neutrophil extracellular trap, *PD* polydatin

To date, increasing evidence has suggested that NETosis is closely associated with the pathogenesis and progression of SLE. In present study, we used two different lupus-prone mouse models to evaluate the effects of PD treatment on SLE development. MRL/*lpr* mice spontaneously develop severe lupus-like features characterized by multiple autoantibodies to nuclear antigens, proteinuria and glomerulonephritis. Here, we treated MRL/*lpr* mice with PD at the age of 12 weeks and continued PD treatment for another 8 weeks. Importantly, PD treatment resulted in a dramatic reduction in proteinuria at the 20 weeks of age (Fig. 2a). MRL/*lpr* mice showed high levels of circulating anti-dsDNA and anti-Sm antibodies, similarly PD treatment markedly reduced those autoantibody levels (Fig. 2b). In addition, kidney H&E staining revealed that Austin scores of kidneys were significantly decreased in PD treated MRL/*lpr* mice (Fig. 2c). Moreover, less deposition of IgG was found in the kidneys of PD-treated mice (Fig. 2d). Particularly, abundant NETs (overlapping DNA and MPO staining) in the glomeruli were easily detected in vehicle-treated MRL/*lpr* mice, but rarely found in PD-treated MRL/*lpr* mice (Fig. 2e). Taken together, these findings suggested that PD treatment could alleviate lupus-like features in MRL/*lpr* mice and reduced the NET formation in the kidney tissue.

### PD treatment also ameliorated lupus manifestations in PIL mice

**Fig. 3:**
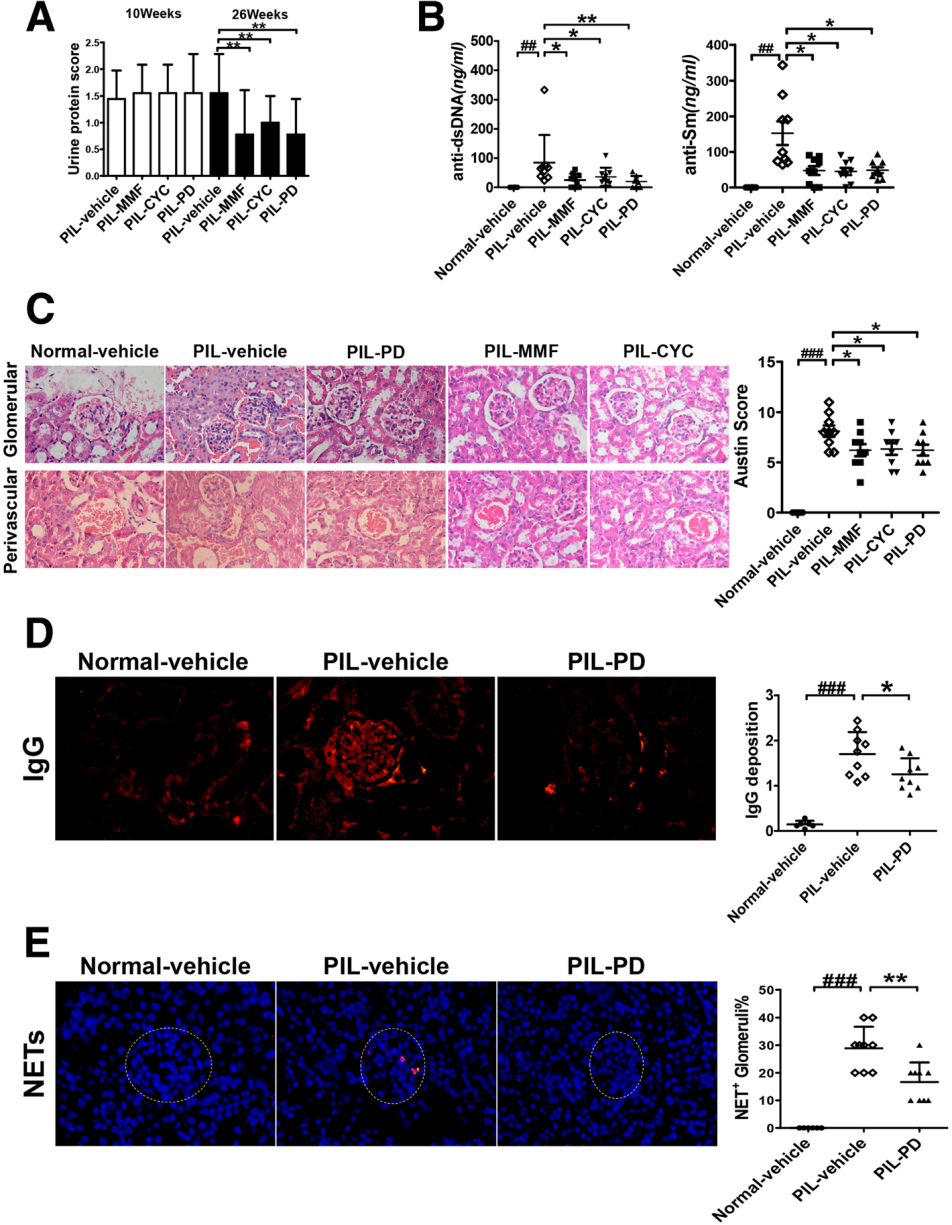
PD ameliorated lupus manifestations and reduced NET deposition in the kidneys in PIL mouse model. PIL mice were treated with vehicle or with PD (45 mg/kg, daily), CYC (1.8 mg/mouse, weekly), MMF (100 mg/kg, daily) for 16 weeks. **a** The proteinuria was assessed as described in Materials and Methods. **b** The levels of anti-dsDNA antibodies and anti-Sm antibodies were examined by ELISA. **c** On the *left*, representative H&E staining of glomerular and renal vascular lesions in kidneys was shown (× 400). On the *right*, Austin scores of kidneys were shown. **d** On the *left*, the glomeruli were stained for IgG deposition and the representative staining images were shown (× 400). On the *right*, 25 glomeruli were analyzed and the average score was calculated for each kidney as described in Materials and Methods. **e** On the *left*, representative staining images of NET formation in kidneys were shown. Colocalization of DNA (*blue*) and MPO (*red*) in kidneys was consistent with NET formation (× 400). On the right, the percentage of NET-stained glomeruli per animal was determined. For all experiments, N = 6 for normal mice; *N* = 9 for each group of treated PIL mice; data were shown as Mean ± SD, *p < 0.05; **p < 0.01; ***p < 0.001. *CYC* cyclophosphamide, IgG immunoglobulin G, *MMF* mycophenolate mofetil, *PD* polydatin, *PIL* pristane-induced lupus

Mycophenolate mofetil (MMF) and cyclophosphamide (CYC) are commonly accepted and effective treatments for lupus nephritis, so we chose these two drugs as positive controls to further investigate the effects of PD treatment on SLE mice. As expected, both MMF and CYC treatments could significantly reduce proteinuria levels, autoantibody levels and Austin activity scores (Fig. 3a, b and c). Similarly, we also found that PD treatment reduced lupus-associated manifestations in PIL mice and decreased NET deposition in the kidneys. However, there were no significant differences in proteinuria levels, autoantibody levels and Austin activity scores among PD, MMF and CYC groups. Compared with vehicle-treated PIL mice, proteinuria levels were significantly decreased in PD-treated PIL mice (Fig. 3a). Furthermore, dramatically decreased levels of serum anti-dsDNA and anti-Sm antibodies were also found in PD-treated PIL mice (Fig. 3b). Additionally, PD treatment markedly reduced the Austin scores and IgG deposition in kidneys (Fig. 3c and d). Finally, we also found that there was a significant decrease in NET formation in the glomeruli of PD-treated PIL mice (Fig. 3e).

### Treatment of PIL mice with PD prevented spontaneous NET formation in vivo

**Fig. 4:**
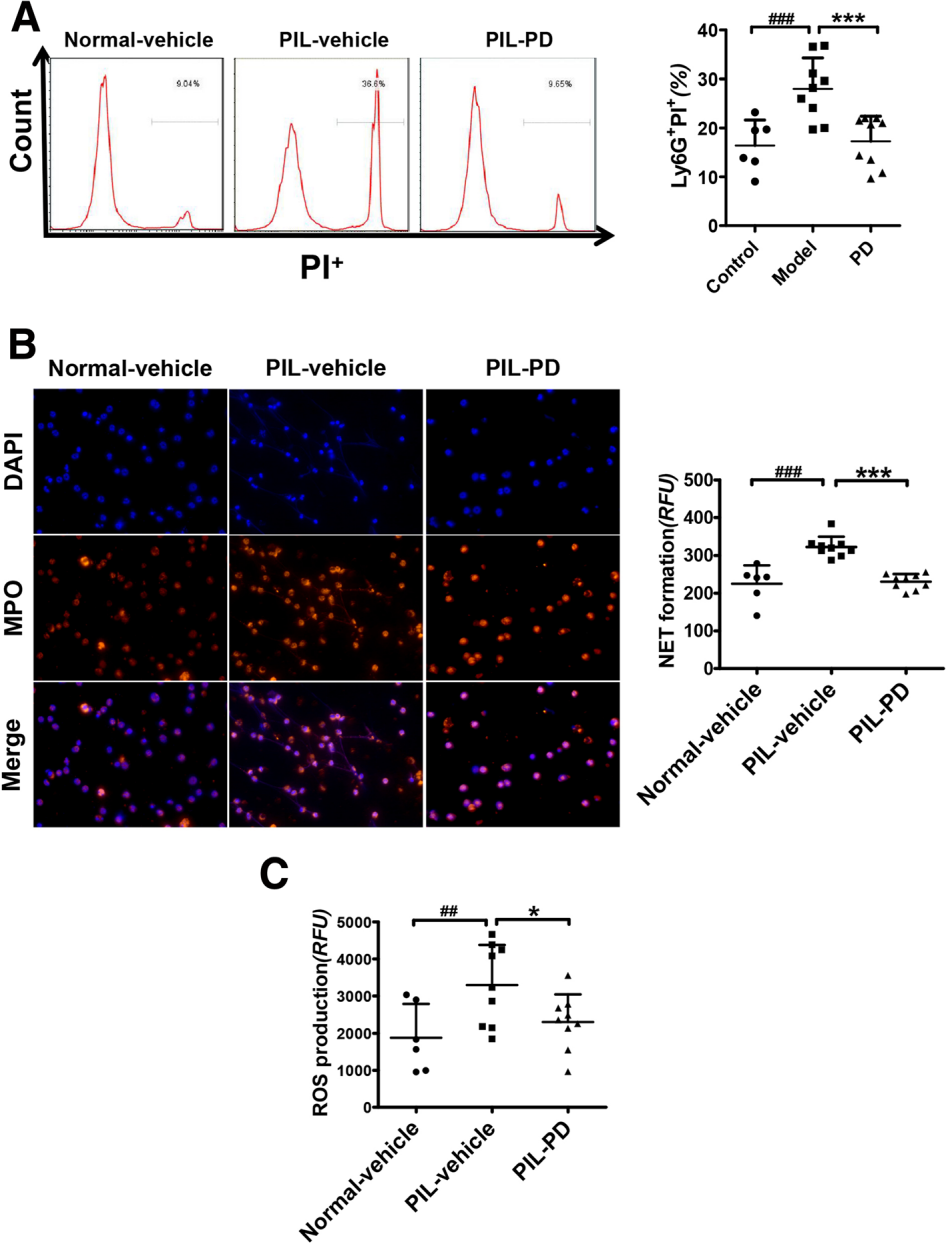
In vivo, PD prevented spontaneous NET formation in pristane-induced lupus mice through inhibition of ROS. **a** After eliminating erythrocytes, the remaining cells from the mouse peripheral blood were incubated in RPMI-1640 with 5% fetal bovine serum. The percentage of Ly6G^+^PI^+^ cells in mouse peripheral blood was tested by flow cytometry. **b** Bone marrow-derived neutrophils from mice were cultured in the absence of serum for 4 h and then the spontaneous NET formation measured. **c** ROS production by the bone marrow-derived neutrophil was measured. For all experiments, data were represented as mean ± SD, ^#^*p* < 0.05; ^##^*p* < 0.01; ^###^*p* < 0.001 were for comparisons between vehicle-treated PIL mouse model and vehicle-treated normal mice; *p < 0.05; **p < 0.01; ***p < 0.001 compared the vehicle and PD-treated PIL mice. *DAPI* 4′, 6′-diamidino-2-phenylindole, *MPO* myeloperoxidase, *NET* neutrophil extracellular trap, *PD* polydatin, *PIL* pristane-induced lupus

Since PD treatment significantly reduced the NET deposition in kidneys of lupus-prone MRL/*lpr* mice, we further identified whether PD treatment could also inhibit NET formation by neutrophils from lupus-prone mice in vivo. Because NETosis is a form of cell death with membrane rupture, we analyzed the percentage of necrotic cells in peripheral blood-derived neutrophils with flow cytometry, illustrated by the percentage of Ly6G^+^PI^+^ cells. As shown in Fig. 4a, necrotic cells were notably decreased in neutrophils treated with PD. Then, we detected for spontaneous NET release by bone marrow-derived neutrophils. As expected, spontaneous NET formation was significantly increased by bone marrow-derived neutrophils from PIL mice, but was inhibited by PD treatment (Fig. 4b). In addition, PD treatment effectively prevented the ROS production by bone marrow-derived neutrophils (Fig. 4c). Collectively, our findings demonstrated that PD treatment suppressed NET formation in pristane-induced lupus mice possibly by reducing ROS generation.

## Discussion

Studies have demonstrated that NETosis plays a significant role in the pathogenesis and progression of SLE. NETosis is recognized as a specific cell death of neutrophils. Based on our prior studies [30, 31], we proposed a “cell death recognition model” for the immune system: consequence of immune responses depends on the ways of cell death. This “cell death” recognition model may well explain the role of NETosis in SLE pathogenesis. During the process of NETosis, intracellular components are released into extracellular space, leading to the presentation of autoantigens to host immune system and the release of damage-associated molecular patterns (DAMPs) that amplify inflammatory and immune responses. It is worth mentioning that peptidylarginine deiminase (PAD) inhibition limited lupus-related skin, renal and vascular damage by preventing NET formation in various lupus-prone mouse models [32, 33]. Moreover, a Janus kinase (JAK) inhibitor, tofacitinib, disrupted NET generation and displayed therapeutic capacity for lupus activity and lupus-related vascular damage [34]. Therefore, modulation of NETosis would be a potential therapeutic avenue for SLE. Consistent with previous studies, in this study we also identified that neutrophils from SLE patients were more prone to undergo NETosis, emphasizing the importance of blocking NETosis for SLE treatment.

During NETosis, ROS generated by nicotinamide adenine dinucleotide phosphate (NADPH)-oxidase mediates the activation of protein-arginine deiminase 4 (PAD4), resulting in decondensation of chromatin and the loss of the nuclear membrane [35]. Subsequently, the decondensed chromatin decorated with cytoplasmic and granule components are released to the extracellular space [35]. It has been shown that inhibiting intracellular or mitochondiral ROS production could effectively prevent NETosis [10, 19]. Thus, blocking ROS production may be of importance for reducing NETosis and alleviating SLE disease activity.

Therefore, in this study, we investigated the effects of PD on ROS-mediated NET formation and further determined therapeutic effects of PD on lupus-prone mice. We first examined whether PD could reduce ROS production by neutrophils. It is well-known that PD exhibits powerful anti-oxidative properties by the inhibition of ROS release by various kinds of cells, such as umbilical vein endothelial cells [32], cardiomyocytes [36] and podocytes [37]. However, whether PD could suppress ROS production by immune cells, especially neutrophils, is still obscure. In this study, it was found that increased intracellular ROS production by human neutrophils was abrogated by PD treatment. Also, PD treatment markedly inhibited ROS production by bone marrow-derived neutrophils from PIL mice. These results demonstrated that PD could prevent ROS production by neutrophils and suggested that PD might inhibit NET formation through blocking ROS production.

Next, we demonstrated the suppressing effects of PD on NET formation. Importantly, we found that PD significantly reduced PMA-induced NET formation by SLE neutrophils and HC neutrophils in vitro. And we detected the percentage of Ly6G^+^PI^+^ cells in peripheral blood of PIL mice and found that PD treatment prevented neutrophils from necrosis, indirectly implying that NETosis was inhibited by PD treatment in vivo. More directly, PD treatment reduced spontaneous NET formation by bone marrow-derived neutrophils. To our knowledge, this is the first report that PD inhibits NET formation both in vivo and in vitro. Therefore, these findings combined with previous results indicate that PD exhibits inhibitory effects on NET formation by reducing ROS production and provide a new approach to modulation of NETosis for SLE treatment.

The combination of pristane-induced lupus model, an environmental factor-induced lupus mouse model, with MRL/*lpr* model, one of spontaneous murine lupus models, can best mimic human lupus, which is mostly affected by environment factors and genetic susceptibility [38]. Therefore, we used two lupus-prone models to assess the effects of PD on SLE pathogenesis and development. As expected, we found that PD effectively ameliorated lupus-like features in MRL/*lpr* mice, showed by the reduction in levels of circulating anti-dsDNA and anti-Sm antibodies, and the improvement in pathology of kidneys. To better mimic the effect of drugs on the development of human lupus clinically, we chose Balb/c mice that had developed proteinuria for further experiments. To our surprise, PD treatment exhibited the same capacity for ameliorating lupus-like features in PIL mice as traditional therapeutic agents targeting T and B lymphocytes (like MMF and CYC) did. Thus, these results confirmed that PD had high therapeutic effect for lupus-prone mice. For further revealing the mechanisms involved in the therapeutic effects of PD on SLE, we investigated whether PD treatment could prevent NET formation by neutrophils and decrease the deposition of NETs in kidneys in vivo. As a result, PD treatment significantly inhibited NET formation by bone marrow-derived neutrophils and reduced the deposition of NETs in kidneys. All the above investigations demonstrated that PD effectively ameliorated lupus-like manifestations in lupus-prone mice perhaps via abrogating NET formation.

A previous study showed that PD treatment limited the symptoms of collagen-induced arthritis for its anti-oxidative and anti-inflammatory effects [26]. Some studies have identified increased levels of NETs in peripheral blood, synovial fluid and rheumatoid nodules of RA patients, suggesting that NET formation may also be correlated with RA [39, 40]. Therefore, our results may provide evidence for an alternative mechanism of PD treatment for rheumatoid arthritis.

## Conclusions

Our study clearly demonstrated that PD significantly blocked ROS-mediated NET formation and effectively attenuated many lupus-like manifestations in two lupus-prone mouse models. These results indicate that PD may have potential clinical values in treating SLE or other autoimmune diseases.

## Abbreviations

CYC: Cyclophosphamide
DAMP: Damage-associated molecular pattern
DAPI: 4′, 6′-diamidino-2-phenylindole
DCFH-DA: 2′,7′-Dichlorodihydrofluorescein diacetate
ELISA: Enzyme-linked immunosorbent assay
H&E: Hematoxylin and eosin
HC: Healthy controls
IBS: Irritable bowel syndrome
IgG: Immunoglobulin G
IL: Interleukin
JAK: Janus kinase
MMF: Mycophenolate mofetil
MPO: Myeloperoxidase
NAC: N-acetyl cysteine
NADPH: Nicotinamide adenine dinucleotide phosphate
NET: Neutrophil extracellular trap
PAD: Peptidylarginine deiminase
PBS: Phosphate-buffered saline
PD: Polydatin
PIL: Pristane-induced lupus
PMA: Phorbol 12-myristate 13-acetate
ROS: Reactive oxygen species
RPMI-1640: Roswell Park Memorial Institute 1640
SLE: Systemic lupus erythematosus
